# Modulation of oxygen consumption rate and vascular endothelial growth factor mRNA expression in human malignant glioma cells by hypoxia

**DOI:** 10.1038/sj.bjc.6690328

**Published:** 1999-04

**Authors:** M J Allalunis-Turner, A J Franko, M B Parliament

**Affiliations:** Department of Oncology, University of Alberta; Departments of Experimental Oncology and, Radiation Oncology, Cross Cancer Institute, 11560 University Avenue, Edmonton, Alberta, Canada T6G 1Z2

**Keywords:** VEGF, hypoxia, oxygen consumption rate, glioma, angiogenesis

## Abstract

Cellular responses to hypoxia include modulation of respiration rate and up-regulation of genes which encode for angiogenesis factors. We tested whether human malignant glioma cells vary in their response to hypoxic stress over the range of oxygen concentrations which exist in tumours. In five cell lines tested, decreased oxygen availability resulted in decreased rates of oxygen utilization, however substantial differences in the magnitude of the response were observed. Northern blot analysis was used to study induction of vascular endothelial growth factor mRNA in response to hypoxia. In two cell lines, modest hypoxia increased vascular endothelial growth factor mRNA levels compared with those of aerobic controls. In two additional cell lines, vascular endothelial growth factor mRNA was constituitively expressed under aerobic conditions and was not further increased by hypoxia. These findings demonstrate that differences in the response to hypoxia exist among human malignant glioma cell lines and suggest that therapies designed to exploit tumour hypoxia may have varying effects in tumours with different hypoxic stress responses. © 1999 Cancer Research Campaign


*British Journal of Cancer* (1999) **80**(1/2), 104–109
© 1999 Cancer Research Campaign
Article no. bjoc.1998.0328

### Modulation of oxygen consumption rate and vascular endothelial growth factor mRNA expression in human malignant glioma cells by hypoxia

**MJ Allalunis-Turner[1,2], AJ Franko[1,2] and MB Parliament[1,3]**

[1]Department of Oncology, University of Alberta, and Departments of [2]Experimental Oncology and [3]Radiation Oncology, Cross Cancer Institute, 11560 University Avenue, Edmonton, Alberta, Canada T6G 1Z2

**Summary** Cellular responses to hypoxia include modulation of respiration rate and up-regulation of genes which encode for angiogenesis factors. We tested whether human malignant glioma cells vary in their response to hypoxic stress over the range of oxygen concentrations which exist in tumours. In five cell lines tested, decreased oxygen availability resulted in decreased rates of oxygen utilization, however substantial differences in the magnitude of the response were observed. Northern blot analysis was used to study induction of vascular endothelial growth factor mRNA in response to hypoxia. In two cell lines, modest hypoxia increased vascular endothelial growth factor mRNA levels compared with those of aerobic controls. In two additional cell lines, vascular endothelial growth factor mRNA was constituitively expressed under aerobic conditions and was not further increased by hypoxia. These findings demonstrate that differences in the response to hypoxia exist among human malignant glioma cell lines and suggest that therapies designed to exploit tumour hypoxia may have varying effects in tumours with different hypoxic stress responses.

**Keywords:** VEGF; hypoxia; oxygen consumption rate; glioma; angiogenesis

Human tumours frequently contain hypoxic regions which develop as a consequence of inadequate blood supply to rapidly growing tissue with metabolic demands. In response to hypoxic stress, tumour cells have been shown to activate a variety of rescue mechanisms, including the up-regulation of genes which code for angiogenesis factors, transcription factors, glucose transporters and glycolytic enzymes (Hochachka et al, 1996). The ability to develop new blood supply and to utilize glucose as an energy source increases the probability of tumour cell survival under oxygen-limiting conditions. Among normal tissues, the production of erythropoietin by the kidney and liver in response to hypoxia is the most familiar physiological example of oxygen-regulated gene expression. Normal tissues also respond to hypoxia by regulating the rate at which oxygen is consumed. Skeletal muscle and normal brain provide the best examples of the variation in response to hypoxic stress which exists among normal tissues. Skeletal muscle decreases oxygen consumption rate as the supply of oxygen becomes limiting, giving rise to the designation of muscle as an ‘oxygen-conforming’ tissue (Hochachka and Guppy, 1987). In contrast, the normal brain continues to consume oxygen at a steady rate which is independent of oxygen availability over a broad range of oxygen concentrations. Normal brain is thus an ‘oxygen-regulatory’ tissue (Hochachka and Guppy, 1987). The oxygen consumption rates of tumours have been examined in vivo. In general, $^{15}O_2$-PET studies show lower extraction ratio for oxygen in gliomas than in the contralateral, normal hemisphere (Lammertsma et al, 1985; Brooks, 1990).

Human malignant gliomas are the most common brain tumour among adults. Necrotic regions within the tumour mass are diagnostic of high-grade gliomas and are believed to arise from microregional exhaustion of oxygen and/or other nutrients. In our previous studies of human malignant glioma cell lines grown as xenografted tumours and labelled in vivo with the hypoxia marker [$^3$H]misonidazole, we observed that a significant proportion of the necrotic regions in these tumours were not associated with hypoxia (Parliament et al, 1997). It is generally accepted that the relative oxygen concentration at any point ‘p’ in a tumour is determined by the supply of oxygen from a functional capillary and the rate at which oxygen is consumed by the cells which lie along the path of oxygen diffusion to ‘p’ (see, for example, Dewhirst et al, 1994). To explain the variable presence of hypoxic regions adjacent to necrosis, we proposed a model in which the oxygen consumption rate of tumours conforms to either ‘oxygen regulatory’ or ‘oxygen conforming’ phenotypes. In the oxygen regulatory model, malignant glioma cells maintain a relatively constant rate of oxygen consumption at all points along the path of diffusion of oxygen from the blood supply, leading to the development of an oxygen gradient as one moves distally to the blood supply. As oxygen becomes a limiting substrate, regions of hypoxia develop at points distant from the vasculature and cell death and necrosis occurs when the oxygen supply is exhausted. This model is consistent with the concept that gliomas utilize oxygen in a manner similar to that of normal brain, and is supported by our previous observations of hypoxic regions adjacent to necrosis in tumour xenografts (Parliament et al, 1997). However, in some regions of xenografted tumours, a pattern of tumour necrosis in the absence of hypoxia suggested that malignant gliomas can also behave as oxygen conformers. Under this condition, diminished oxygen supply to tumour cells distant from blood vessels may be detected intracellularly by an ‘oxygen sensor’ (Chandel et al, 1997), resulting in a number of signalling events including ultimately decreased respiration. Because less oxygen is consumed, its diffusion distance is increased and hypoxic regions, when they occur, develop

*Received 19 December 1997*
*Revised 3 July 1998*
*Accepted 4 August 1998*

*Correspondence to:* MJ Allalunis-Turner at relatively greater distances from the blood supply than in the oxygen regulatory model. Cellular necrosis develops, but as a consequence of the exhaustion of some essential nutrient(s) other than oxygen (e.g. glucose). To determine whether this model is correct, we selected human glioma cells which exhibit different patterns of hypoxia adjacent to necrosis, and measured both oxygen consumption rate and vascular endothelial growth factor (VEGF) gene expression under in vitro conditions which permitted the concentration of oxygen in the atmosphere to be precisely regulated.

### MATERIALS AND METHODS

### Cell lines and xenografts

Details of the origin and characterization of the glioma cell lines used in this study have been previously published (Allalunis-Turner et al, 1992; Parliament et al, 1997). All animal experiments were performed according to guidelines for the use of animals in research established by the Canadian Council for Animal Care. Details of the initiation and growth of tumour xenografts were as previously described (Parliament et al, 1997).

### Hypoxic cultures and oxygen consumption rate

To study the effect of changes in oxygen tension on oxygen consumption rate and vascular endothelial growth factor (VEGF) expression, a gas-exchange manifold system composed of a series of leak-proof aluminium chambers (Koch et al, 1979) was used to create atmospheres of varying proportions of oxygen in 5% carbon dioxide/95% nitrogen. Cells were trypsinized from exponentially growing monolayer cultures, seeded onto glass plates and incubated under standard conditions (18% oxygen) or in aluminium chambers under moderately hypoxic conditions corresponding to 2% or 0.6% oxygen. Sealed chambers were incubated at 37°C for 4 days. At the end of the incubation period, plates were removed from the chambers and the cells were trypsinized, washed and resuspended in complete medium. The oxygen consumption rate was determined using a Clark-type electrode (Koch, 1984) which measured the oxygen concentration continuously in the cell suspension in a specially designed spinner chamber held in a 37°C water bath. The oxygen consumption was calculated from the linear portion of the oxygen consumption curve and was determined within the first hour after electrode stabilization. In other experiments, a colony-forming assay was used as previously described (Allalunis-Turner et al, 1992) to assess the proportion of clonogenic cells recovered after growth under hypoxic conditions.

### Northern blotting

Total RNA was isolated from cells cultured for 5 h under aerobic or hypoxic conditions using the guanidinium/silica gel column method (Qiagen). Twenty micrograms of total RNA was electrophoresed in each lane. After transfer, the blots were hybridized with $^{32}$P-random-labelled probes to β-actin or VEGF (a kind gift from Dr Harold Dvorak). The 204-bp human VEGF probe was originally prepared by reverse transcription polymerase chain reaction (PCR) with oligonucleotide primers [(forward): 5′-CGCGGATCCAGGAGTACCCTGATGAG-3′; (reverse) 5′-CCGGAATTCACATTTGTTGTGCTGT-3′] based on the human VEGF sequence (Berse et al, 1992). This probe was designed specifically to recognize all VEGF transcripts and does not discriminate among alternatively spliced transcripts (Berse et al, 1992). To quantify changes in VEGF mRNA expression, the radiographic film used to detect $^{32}$P decay was scanned using a PDQUEST densitometer (PDI, Huntington Station, NY, USA). Optical densities of the regions of the film corresponding to the VEGF and β-actin bands were determined using PDI Quantity One scanning and analysis software. VEGF mRNA optical densities were normalized using β-actin mRNA of each samples as a control. Increases in mRNA after exposure to hypoxic conditions are expressed as a percentage of the aerobic (18% oxygen) cultures.

### Immunohistochemistry

Cells were grown for 24 h in aluminium chambers in atmospheres of 18%, 2% or 0.6% oxygen as described above. After trypsinization and washing, cells were spun onto glass microscope slides (Shandon Cytospin) and fixed with methacarn, a solution of absolute methanol (60%), chloroform (30%) and glacial acetic acid (10%). A polyclonal antibody (Santa Cruz), which recognized the 121, 165 and 189 isoforms of VEGF, and the Fast Red detection system (BioGenex) were used to analyse VEGF protein expression. A cell showing one or more foci of intense red staining was judged to be positive for VEGF protein expression. For each cell line, ≥ 500 cells were examined and the percentage of cells staining positive for VEGF expression was determined.

### Statistics

A computer based *t*-test (StatView for the Mac) was used to evaluate the significance of differences observed.

### RESULTS

### Oxygen consumption rates

The effect of moderate hypoxia on oxygen consumption rate was determined for human glioma cells which exhibit different patterns of hypoxia adjacent to necrosis in vivo. Figure 1A plots the oxygen consumption rates as a function of percentage of oxygen in the gas phase of the incubation chambers. Included in this figure for reference are the oxygen consumption rates previously determined for xenograft explants established from these same glioma cell lines (Parliament et al, 1997). For four parental (M006 and M059K) and xenograft-derived (M006x and M059Kx) cell lines, the mean values of the oxygen consumption rates measured under fully aerobic conditions (18% oxygen) were significantly greater ($P < 0.05$) than the mean values determined for cells incubated in atmospheres of 2% or 0.6% oxygen for 4 days. In contrast, the mean oxygen consumption rate of M010b cells showed a greater variability in response to reduced oxygen, with no values measured under hypoxic conditions being statistically different from that of the aerobic controls.

To facilitate comparisons, the oxygen consumption rate of each cell line was normalized to that previously determined for the corresponding tumour xenograft explant (Figure 1B). Cells grown under standard incubator conditions (18% oxygen) consumed oxygen at rates 2.4- to 4.8-fold greater than those measured for freshly dissociated tumour explants. When cells grown in 18% oxygen were transferred to modestly hypoxic conditions for 4 days, oxygen consumption rates were reduced. The oxygen consumption rates of cells incubated at 2% oxygen differed from those of the corresponding tumour explants by factors of 0.4–2.4. At 0.6% oxygen, the oxygen consumption rates approached those of the tumour explants (0.5–1.4).

© Cancer Research Campaign 1999

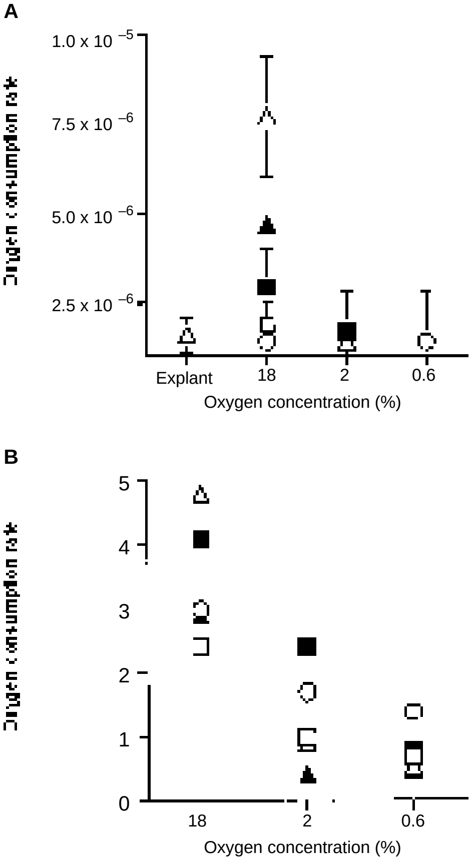

**Figure 1 (A)** Oxygen consumption rates (µMeell$^{-1}$ h$^{-1}$) of malignant glioma cells grown in atmospheres of 18%, 2% or 0.6% oxygen. **A**: Results from four or more replicate experiments are expressed as means ± s.d.; ○ = M010b; □ = M006; ■ = M006x; △ = M059K; ▲ = M059Kx. Oxygen rates previously determined for tumour xenograft explants (Parliament et al, 1997) are provided for comparison. When fewer than five points are shown, the symbols overlap, and only one (or two) symbols are shown. The following *P*-values were obtained when the differences in mean oxygen consumption rate of cells incubated under 18% vs. 2% oxygen and 18% vs. 0.6% oxygen, respectively, were compared: M006, $P < 0.03$, $P < 0.02$; M006x, $P < 0.03$, $P < 0.003$; M059K, $P < 0.02$, $P < 0.005$; M059K×, $P < 0.02$, $P < 0.004$; M010b, $P > 0.1$, $P > 0.1$. (**B**) The oxygen consumption rate of each cell line shown in **A** was normalized to that previously determined for the corresponding tumour xenograft explant

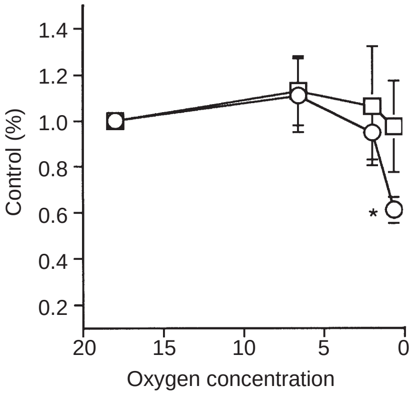

**Figure 2** Colony-forming cells recovered from cultures of M010b (○) or M006 (□) cells grown in atmospheres of 18%, 2% and 0.6% oxygen. Results from four or more replicate experiments are expressed as means ± s.d. and are provided as a percentage of the 18% oxygen value. *$P < 0.01$

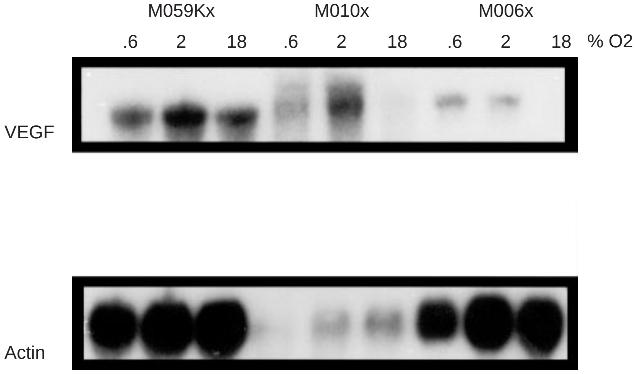

**Figure 3** Northern blot analysis of VEGF mRNA expression in M059Kx, M010bx and M006x glioma cells exposed for 5 h to atmospheres of 18%, 2% or 0.6% oxygen

To determine whether the decrease in oxygen consumption rate was the result of cell death, the clonogenic cell survival of M006 and M010b cells after exposure to 4 days of moderate hypoxia was determined (Figure 2). Compared with control cells maintained at 18% oxygen, exposure of M010b cells to moderate hypoxia (0.66% oxygen) resulted in a significant ($P < 0.01$) decrease in the proportion of clonogenic cells, despite the ability of M010b cells to maintain a relatively steady rate of oxygen consumption. In contrast, hypoxia did not reduce the proportion of clonogens in the M006 cell line, which significantly decreased oxygen utilization in response to hypoxia.

### VEGF mRNA expression

We tested whether oxygen regulates VEGF mRNA expression over the range of concentrations likely to exist in human tumours and whether this expression differs in glioma cells which vary in their ability to reduce cellular respiration in response to hypoxia. Low levels of VEGF mRNA were observed in M006 and M010b cells cultured under fully aerobic conditions (Figure 3). Under hypoxic conditions, an eight- to tenfold increase in VEGF message was observed for both cell lines. In contrast, VEGF message was constituitively expressed in aerobic M059K cells and was not further increased by hypoxia.

To address the question of whether chronic exposure to hypoxia can alter the expression of hypoxia-responsive genes, M006× tumour spheroids were cultured at 0.6% oxygen for 13 days, giving rise to the cell line designated M006×Lo. In comparison with the parental line (M006×) in which moderate hypoxia increased VEGF message ~15-fold, M006×Lo cells showed constitutive VEGF mRNA expression which was only slightly (i.e. 1.5 – twofold) increased by hypoxia (Figure 4).

### VEGF protein expression

To assess whether differences in VEGF mRNA expression observed among the three glioma cell lines reflected differences in protein expression, immunohistochemical staining of VEGF protein was performed using cells maintained under modestly hypoxic conditions for 24 h. Compared with aerobic control, exposure of M010bx, M006x and M059Kx cells to atmospheres of 2% oxygen (Figure 5) or 0.6% oxygen (data not shown) resulted in an increased percentage of cells staining positive for VEGF protein expression. The percentage of VEGF-positive cells under conditions of 18%,

 *© Cancer Research Campaign 1999*

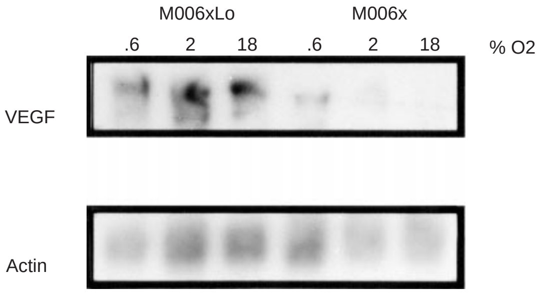

**Figure 4** Northern blot analysis of VEGF mRNA expression in parental (M006x) and hypoxia-adapted (M006xLo) glioma cells exposed for 5 h to atmospheres of 18%, 2% or 0.6% oxygen

2% or 0.6% oxygen, respectively, were M010bx (21%, 75%, 62%), M006x (17%, 97%, 92%) and M059Kx (60%, 99%, 56%). The relatively increased (60%) positive staining for VEGF observed in aerobic M059Kx cells was consistent with the observed constitutive aerobic expression of VEGF mRNA in this cell line.

### DISCUSSION

We have previously shown that the oxygen consumption rates of human malignant glioma cell lines grown as xenografts, in which the $pO_2$ values would be expected to range between roughly 0.2% and 8%, were significantly lower than those determined for the same cell lines grown in vitro in which the oxygen in the medium was in near equilibrium with that of the atmosphere (18% oxygen) (Parliament et al, 1997). This suggested that malignant glioma cells may possess a functional oxygen sensor that allows the rates of oxygen consumption to conform to oxygen availability in a manner similar to that reported for normal hepatocytes. To test this hypothesis, we measured oxygen consumption rates of human malignant glioma cell lines after growth in vitro under fully aerobic (18% oxygen) or modestly hypoxic (2% and 0.6% oxygen) conditions. In five tumour cell lines tested, decreased oxygen availability resulted in decreased rates of oxygen utilization, although the magnitude of the response varied greatly among the cell lines. The decreased oxygen consumption rates were not attributable to loss of cell viability because on average >90% of the cells recovered from the incubation chambers were viable as assessed by a dye exclusion assay (data not shown). These results are in marked contrast to previous studies of the oxygen dependence of cellular respiration. Several investigators have shown that the rate of oxygen consumption remains independent of oxygen tension down to very low levels of oxygen ($K_m < 1\ \mu M$) (Froese, 1962; Boag, 1970; Erecinska and Wilson, 1982). However, the experimental design of the present work differs from previous studies in that cells were maintained under hypoxic conditions for 4 days before determination of oxygen consumption rate. In earlier studies, oxygen consumption rates were determined for cells maintained in

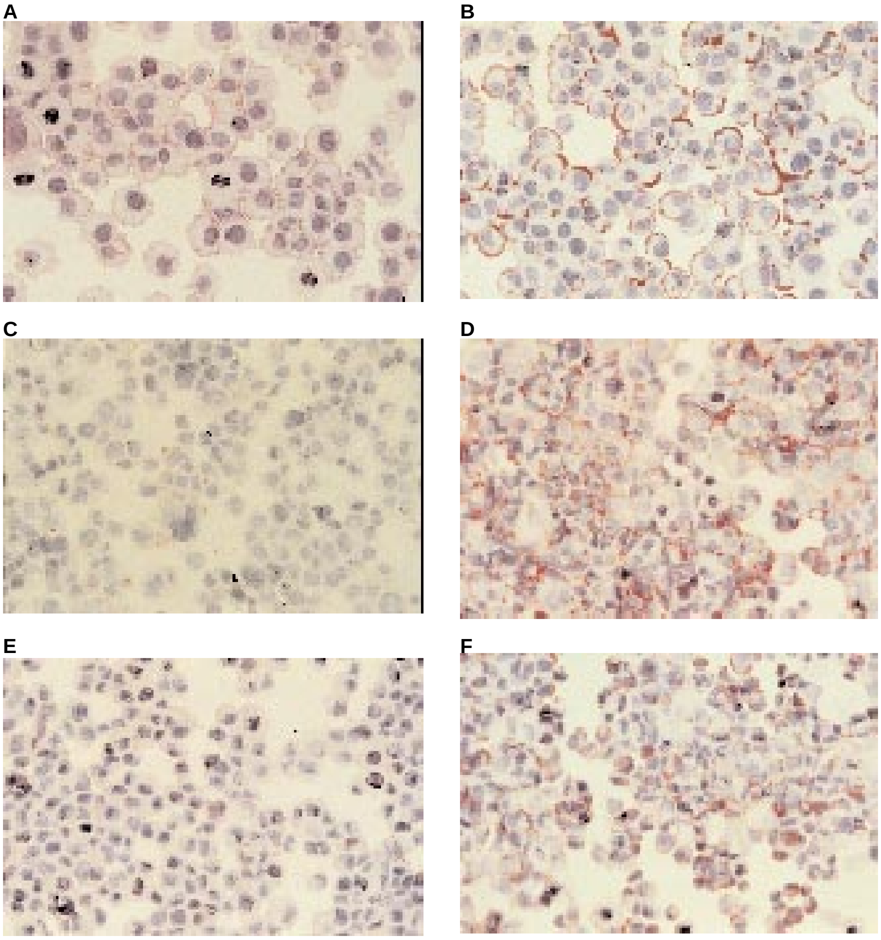

**Figure 5** Immunohistochemical analysis of VEGF protein expression in human malignant glioma cells cultured for 24 h under aerobic or hypoxic conditions. (**A**) M059Kx, 18% oxygen; (**B**) M059Kx, 2% oxygen; (**C**) M010bx, 18% oxygen; (**D**) M010bx, 2% oxygen; (**E**) M006x, 18% oxygen; (**F**) M006x, 2% oxygen closed systems for a few hours. The extended hypoxic incubation period used in our studies may have allowed for the activation of a cascade of hypoxic defence mechanisms. In addition, the response to hypoxic stress in malignant glioma cells may be intrinsically different from that of tumour cells grown as ascites.

As an initial response to oxygen deficit, hypoxia-tolerant cells suppress cellular ATP-demand and -utilization pathways (Hochachka et al, 1996). In recent studies, Chandel et al (1997) have shown that normal hepatocytes regulate their cellular respiration as a function of oxygen availability, with reduction of oxygen utilization under physiological levels of hypoxia and restoration of basal levels upon reoxygenation. Further, the absence of a significant increase in lactate production during hypoxic incubation suggested that anaerobic ATP production did not contribute to the energy supply. For hepatocytes, reversible changes in the $V_{max}$ of cytochrome *c* oxidase in response to hypoxia suggests that cytochrome *c* oxidase may function as an oxygen sensor. The putative oxygen sensor in tumour cells remains to be identified, however cytochrome *c* oxidase or other members of the electron transport chain are likely candidates.

The ability to regulate cellular respiration could confer a significant survival advantage for tumour cells. Studies by Dewhirst et al (1994) have provided evidence that such regulation may exist in tumours in situ. Using a rat mammary adenocarcinoma model, they have shown that significant variations in oxygen consumption occur at the microregional level, suggesting that tumour cells can modulate cellular respiration in response to local variations in oxygen availability. Our in vitro results showing reduced oxygen consumption in response to hypoxia are consistent with the studies of Dewhirst et al (1994), and provide additional support for the hypothesis that microregional control of cellular respiration is a component of the hypoxic stress response.

In principle, the ability to modulate tumour cell respiration would provide a mechanism to improve the therapeutic response to ionizing radiation and certain chemotherapeutic agents. Using a theoretical simulation of oxygen transport in a rat tumour model, Secomb et al (1995) have calculated that 30% reduction in oxygen consumption rate would be sufficient to eliminate tumour hypoxia. The data presented in this study suggest that: (i) the ability of malignant glioma cell lines to modulate their rates of respiration varies considerably; and (ii) reductions in oxygen consumption rate calculated for in vitro cell cultures may not adequately predict the changes likely to occur in vivo. As regards the first point, the baseline oxygen consumption rates of the cell lines grown under standard incubator conditions varied by factor of 5. The magnitude of the difference in oxygen consumption rate observed when cells were moved from aerobic to hypoxic conditions was greatest for the cell lines M059K and M059Kx, which have the greatest baseline oxygen consumption rates. Nonetheless, the theoretical target of 30% reduction in oxygen consumption rate vis-à-vis that of aerobic controls was achieved even for the cell line with the lowest baseline oxygen consumption rate (M010b). However, if one were to use changes in respiration rate as a means of assessing the feasibility of reduction of oxygen consumption rates of tumours in situ, then measurement of consumption rates relative to the baseline values of tumour explants may provide a more relevant analysis. For the glioma cells in this study, the baseline oxygen consumption rates of freshly dissociated tumour explants varied by a factor of 3.5. As was the case for cells grown under standard incubator conditions, the M059K tumour explants showed the greatest rate of oxygen consumption and the M010b tumour explants the least. When M059K cells were cultured under hypoxic conditions, their oxygen consumption rate was reduced to approximately one-half the value of that of the tumour explant. In contrast, hypoxia (2% and 0.6% oxygen) was unable to reduce the oxygen consumption rate of the M010b cell line relative to the baseline value established for M010b tumour explants. Moreover, exposure of M010b cells to hypoxia (0.6% oxygen) resulted in a significant loss of colony-forming ability. Thus, it is unlikely that the failure to reduce oxygen consumption rate below that of the tumour explant was due to insufficient hypoxic stress. These results suggest that strategies to improve tumour oxygenation by reducing oxygen consumption rate may not be effective in tumours which have, on average, a relatively low rate of cellular respiration.

Overall, the variable patterns of oxygen utilization described for these glioma cell lines in vitro are consistent with those observed for the same cells grown as xenografted tumours. In M010b xenografts, virtually all areas of necrosis were associated with severe hypoxia (Parliament et al, 1997). In contrast, in M006 and M059K tumours, ~20–50% of necrotic regions showed no evidence of severe hypoxia. This suggests that the M006 and M059K cell lines contain subpopulations of tumour cells capable of modulating oxygen consumption in vivo, whereas subpopulations of cells with such properties may be less abundant in the M010b cell line. Although in vitro determinations of oxygen consumption rates are useful in identifying overall responses to hypoxic stress, such measurements will fail to detect tumour subpopulations which may differ in their patterns of oxygen utilization. To address these questions, experiments to identify intratumoural differences in response to hypoxia are currently underway using clonally derived cells.

The ability to up-regulate angiogenesis factors is a critical component of a tumour's ability to respond to hypoxic stress by developing new vasculature supply (reviewed in Hanahan and Folkman, 1996). VEGF is a potent angiogenic factor (reviewed in Ferrara et al, 1992; and in Dvorak et al, 1995). In human cells, four alternatively spliced transcripts encode polypeptides of 121, 165, 189 and 201 amino acids. $VEGF_{121}$ and $VEGF_{165}$ are secreted in soluble form and promote endothelial cell mitogenesis. $VEGF_{185}$, $VEGF_{206}$ and a percentage of $VEGF_{165}$ polypeptides are secreted but remain bound to the cell surface, possibly serving as a reservoir of biologically active VEGF (Houck et al, 1992). Among human gliomas, $VEGF_{165}$ is the predominant form (Berkman et al, 1993). The VEGF gene contains a hypoxia-inducible factor (HIF-1) binding site within the 5′ enhancer region (Ema et al, 1997; Levy et al, 1997) and has been demonstrated to be transcriptionally activated in response to hypoxia (Iliopoulos et al, 1996; Bussolino et al, 1997; Levy et al, 1997). Increased levels of both VEGF mRNA ligand and receptor have been reported in a variety of human cancers (Ohta et al, 1996; Suzuki et al, 1996; Fontanini et al, 1997; Salver et al, 1997) including glioblastoma (Plate et al, 1992, 1994), and may confer a growth advantage and/or be a marker of aggressive tumour behaviour. In this study, we tested whether the induction of VEGF mRNA in response to physiologically relevant hypoxic conditions would differ among glioma cell lines. No apparent correlation between the induction of VEGF expression and the patterns of cellular respiration in response to hypoxic stress was observed as modest levels of hypoxia increased production of VEGF mRNA above that found in aerobic cells in both M006x and M010b cells. The response to hypoxic stress involves activation of multiple pathways (Hochachka et al, 1996). There is no reason to expect that all pathways will respond identically. Our data suggest that the pathways leading to VEGF induction and oxygen consumption are clearly regulated differently during hypoxic stress.

 © Cancer Research Campaign 1999

In this study, we observed differences in the basal level of VEGF transcript and protein and in the magnitude of the induction of VEGF in response to hypoxia. VEGF mRNA was constitutively expressed in M059Kx and M006xLo cells, and its expression was not further increased by hypoxia. White et al (1995) reported high levels of constitutive VEGF message expression in some human tumour cell lines, and have shown that such VEGF-overexpressing cells are least responsive to hypoxia. In their comparison of intracellular VEGF concentrations in the HCT-8 colon carcinoma cell line and its clonal derivative clone A, Leith and Michelson (1995) noted similar differences in both the basal levels of VEGF and in its rate of secretion in response to hypoxia. Mechanisms involving post-transcriptional regulation of VEGF leading to increased message stability are believed to contribute to constitutive elevation of VEGF observed in human tumour cell lines (White et al, 1995; Levy et al, 1996, 1997). Experiments to determine whether the half-life of VEGF mRNA produced by M059Kx and M006xLo cells is modified are currently underway.

In summary, although others have reported the induction of VEGF mRNA in human tumour cell lines grown under severely hypoxic or anoxic conditions, our studies demonstrate that oxygen levels approximating venous $pO_2$ are sufficient to activate VEGF transcription. Further, our results suggest that significant intra- and intertumoral differences in oxygen utilization may exist among human malignant gliomas and that microregional differences in oxygenation may result in heterogeneous transcriptional activation of hypoxia-inducible genes. The influence such differences may have on therapies designed to exploit tumour hypoxia remains an intriguing question.

### ACKNOWLEDGEMENTS

The authors thank Cheryl Santos, Rangu Mandyam and Brenda Wolokoff for technical assistance. This work was supported by the National Cancer Institute of Canada with funds provided by the Canadian Cancer Society.

### REFERENCES

Allalunis-Turner MJ and Siemann DW (1986) Characterization of human tumor xenografts using different enzyme dissociation techniques. *Br J Cancer* **54**: 615–622

Allalunis-Turner MJ, Barron GM, Day RS, III, Fulton DS and Urtasun RC (1992) Radiosensitivity testing of human primary brain tumor specimens. *Int J Radiat Oncol Biol Phys* **23**: 339–343

Berkman RA, Merrill MJ, Reinhold WC, Monacci WT, Saxena A, Clark WC, Robertson JT, Ali IU and Oldfield EH (1993) Expression of the vascular permeability factor/vascular endothelial growth factor gene in central nervous system neoplasms. *J Clin Invest* **91**: 153–159

Berse B, Brown LF, Van De Water L, Dvorak HF and Senger DR (1992) Vascular permeability factor (vascular endothelial growth factor) gene is expressed differentially in normal tissues, macrophages and tumors. *Mol Biol Cell* **3**: 211–220

Boag JW (1970) Cell respiration as a function of oxygen tension. *Int J Radiat Biol* **5**: 475–478

Brooks D (1990) In-vivo metabolism of human cerebral tumors. In *Neuro-Oncology: Primary Malignant Brain Tumors*, Thomas D (ed.), pp. 122–134. Johns Hopkins University Press: Baltimore

Bussolino F, Mantovani A and Persico G (1997) Molecular mechanisms of blood vessel formation. *Trends Biol Sci* **22**: 251–256

Chandel NS, Budinger GRS, Choe SH and Schumaker PT (1997) Cellular respiration during hypoxia. *J Biol Chem* **272**, 18808–18816.

Dewhirst MW, Secomb TW, Ong ET, Hsu R and Gross JF (1994) Determination of local oxygen consumption rates in tumors. *Cancer Res* **54**: 3333–3336

Dvorak HF, Brown LF, Detmar M and Dvorak AM (1995) Vascular permeability factor/vascular endothelial growth factor, microvascular hyperpermeability, and angiogenesis. *Am J Pathol* **146**: 1029–1039

Ema M, Taya S, Yokotani N, Sogawa K, Matsuda Y and Fujii-Kuriyama Y (1997) A novel bHLH-PAS factor with close sequence similarity to hypoxia-inducible factor 1a regulates the *VEGF* expression and is potentially involved in lung and vascular development. *Proc Natl Acad Sci USA* **94**: 4273–4278

Erecinska M and Wilson DF (1982) Regulation of cellular energy metabolism. *J Membr Biol* **70**: 1–14

Ferrara N, Houck K, Jakeman L and Leung DW (1992) Molecular and biological properties of the vascular endothelial growth factor family of proteins. *Endocrinol Rev* **13**: 18–29

Fontanini G, Vignati S, Boldrini L, Chiné S, Silvestri V, Lucchi M, Mussi A, Angeletti AA and Bevilacqua G (1997) Vascular endothelial growth factor is associated with neovascularization and influences progression of non-small cell lung carcinoma. *Clin Cancer Res* **3**: 861–865

Froese G (1962) The respiration of ascites tumor cells at low oxygen concentrations. *Biochim Biophys Acta* **57**: 509–519

Hanahan D and Folkman J (1996) Patterns and emerging mechanisms of the angiogenic switch during tumorigenesis. *Cell* **86**: 353–364

Hochachka PW and Guppy M (1987) Animal anaerobes. In *Metabolic Arrest and the Control of Biological Time*, pp. 10–35. Harvard University Press: Cambridge

Hochachka PW, Buck LT and Land SC (1996) Unifying theory of hypoxia tolerance: molecular/metabolic defense and rescue mechanisms for surviving oxygen lack. *Proc Natl Acad Sci USA* **93**: 9493–9498

Houck KA, Leung DW, Rowland AM, Winter J and Ferrara N (1992) Dual regulation of vascular endothelial growth factor bioavailability by genetic and proteolytic mechanisms. *Biol Chem* **267**: 26031–26037

Iliopoulos O, Levy AP, Jiang C, Kaeling WG Jr and Goldberg MA (1996) Negative regulation of hypoxia-inducible genes by the von Hippel-Lindau protein. *Proc Natl Acad Sci USA* **93**: 10595–10599

Koch CJ (1984) A 'thin film' culturing technique allowing rapid gas-liquid equilibrium (6 seconds) with no toxicity to mammalian cells. *Radiat Res* **97**: 434–438

Koch CJ, Howell RL and Biaglow JE (1979) Ascorbate anion potentiates the cytotoxicity of nitro-aromatic compounds under hypoxic and anoxic conditions. *Br J Cancer* **39**: 321–329

Lammertsma MA, Wise R, Cox T, Thomas D and Jones T (1985) Measurement of blood flow, oxygen utilization, oxygen extraction ratio and fractional blood volume in human brain tumours and surrounding oedematous tissue. *Br J Radiol* **58**: 725–734

Leith JT and Michelson S (1995) Secretion rates and levels of vascular endothelial growth factor in clone A or HCT-8 human colon tumour cells as a function of oxygen concentration. *Cell Prolif* **28**: 415–430

Levy AP, Levy NS and Goldberg MA (1996) Post-transcriptional regulation of vascular endothelial growth factor by hypoxia. *J Biol Chem* **271**: 2746–2653

Levy AP, Levy NS, Iliopoulos O, Jiang C, Kaelin WG Jr and Goldberg MA (1997) Regulation of vascular endothelial growth factor by hypoxia and its modification by the von-Hippel-Lindau tumor suppressor gene. *Kidney Int* **51**: 575–578

Ohta Y, Endo Y, Tanaka M, Shimizu J, Oda M, Hayashi Y, Watanabe Y and Sasaki T (1996) Significance of vascular endothelial growth factor messenger RNA expression in primary lung cancer. *Clin Cancer Res* **2**: 1411–1416

Parliament MP, Franko AJ, Allalunis-Turner MJ, Mielke BW, Santos CL, Wolokoff BG and Mercer JR (1997) Anomalous patterns of nitroimidazole binding adjacent to necrosis in human glioma xenografts: possible role of decreased oxygen consumption. *Br J Cancer* **75**: 311–318

Plate KH, Breier G, Weich HA and Risau W (1992) Vascular endothelial growth factor is a potential tumour angiogenesis factor in human gliomas in vivo. *Nature* **359**: 845–848

Plate KH, Breier G, Weich HA, Mennel HD and Risau W (1994) Vascular endothelial growth factor and glioma angiogenesis: coordinate induction of VEGF receptors, distribution of VEGF protein and possible in vitro regulatory mechanisms. *Int J Cancer* **59**: 520–529

Salver P, Mäenpää H, Orpana A, Alitalo K and Joensuu H (1997) Serum vascular endothelial growth factor is often elevated in disseminated cancer. *Clin Cancer Res* **3**: 647–651

Secomb TW, Hsu R, Ong ET, Gross JF and Dewhirst MW (1995) Analysis of the effects of oxygen supply and demand on hypoxic fraction in tumors. *Acta Oncol* **34**: 313–316

Suzuki K, Hayashi N, Miyamoto Y, Yamamoto M, Ohkawa K, Ito Y, Sasaki Y, Yamaguchi Y, Nakase H, Noda K, Enomoto N, Arai K, Yamada Y, Yoshihara H, Tujimura T, Kawano K, Yoshikawa K and Kamada T (1996) Expression of vascular permeability factor/vascular endothelial growth factor in human hepatocellular carcinoma. *Cancer Res* **56**: 3004–3009

White FC, Carroll SM, and Kamps MP (1995) VEGF mRNA is reversibly stabilized by hypoxia and persistently stabilized in VEGF-overexpressing human tumor cell lines. *Growth Factors* **12**: 289–301

*© Cancer Research Campaign 1999*
